# Left Frontal Lobe Cardioembolic Stroke as a Co-presentation of Bicuspid Aortic Valve With Severe Stenosis and a Basal Interventricular Septal Aneurysm in a Young Female Patient

**DOI:** 10.7759/cureus.49003

**Published:** 2023-11-18

**Authors:** Jenish Bhandari, Shweta Paulraj, Azhar Hussain, Sanchari Banerjee, Ryan D Ruia, Debanik Chaudhuri

**Affiliations:** 1 Internal Medicine, Upstate University Hospital, Syracuse, USA; 2 Internal Medicine, State University of New York Upstate Medical University, Syracuse, USA; 3 Internal Medicine, Norton College of Medicine at Upstate Medical University, Syracuse, USA; 4 Interventional Cardiology, State University of New York Upstate Medical University, Syracuse, USA

**Keywords:** s: magnetic resonance imaging, severe aortic stenosis, interventricular septal aneurysm, cardioembolic stroke, bicuspid aortic valve

## Abstract

Bicuspid aortic valve (BAV) and interventricular septum (IVS) aneurysms are common congenital heart defects affecting 1.3% and 0.3% of the population, respectively. The coexistence of membranous types of IVS aneurysm and BAV is even rarer. We report a case of a 48-year-old woman with a history of BAV and severe aortic stenosis who had a seizure in a grocery store and was brought to the emergency department (ED). An MRI of the brain without contrast revealed a left frontal lobe acute lacunar infarct, suggestive of embolic origin. A transesophageal echocardiogram confirmed a basal IVS aneurysm measuring 12.2 mm × 16 mm without intracardiac shunting or thrombi. We diagnosed her with cardioembolic stroke as a complication of BAV and IVS aneurysm and initiated anticoagulation as she did not qualify for surgical intervention. This report emphasizes that IVS aneurysms associated with BAV, although often asymptomatic, may cause adverse outcomes such as cardioembolic stroke. Therefore, timely detection by non-invasive imaging, including echocardiography, CT scans, and MRI, and appropriate intervention are essential to improving health outcomes and survival.

## Introduction

Bicuspid aortic valve (BAV) and interventricular septum (IVS) aneurysms are common congenital heart defects, affecting 1.3% and 0.3% of the population, respectively [1]. The coexistence of membranous types of IVS aneurysm and BAV is even rarer. Aortic aneurysms, premature aortic valve stenosis, or regurgitation have been reported with BAV [2]. IVS aneurysm is a very uncommon cardiac pathology, with a reported prevalence of around 0.3% of patients with congenital structural heart anomalies [3]. The IVS has two morphological components, i.e., the muscular and membranous portions, and the latter portion is more prone to aneurysms. The co-existence of the membranous IVS aneurysm and bicuspid valve is rare. We report a case of a 46-year-old woman who presented with cardioembolic stroke due to BAV with severe stenosis and IVS aneurysm. A brief version of this case report has been presented at the CHEST 2022 conference [4].

## Case presentation

We report the case of a 46-year-old woman with a history of Hodgkin's lymphoma treated with chemotherapy, ischemic strokes in the right basal ganglia and left cerebellum, and severe aortic stenosis due to a bicuspid aortic valve. She had a seizure in a grocery store and fell to the floor with generalized jerking movements. She was brought to the emergency department (ED) with hypertension (199/109 mmHg) and tachycardia (102 beats per minute) but was otherwise vitally stable (Table 1).

**Table 1:** Important laboratory parameters of the case

Parameter	Value	Normal range
White blood cell (10^9^/L)	18.70	4.5–11
Anion gap (mmol/L)	21	8-12
Beta-hydroxybutyrate, serum (mmol/L)	1.99	0.4–0.9
Hemoglobin A1c (%)	11.3	<6.4

She was found to have a defective insulin pump. She had another tonic-clonic seizure in the ED and received lorazepam and levetiracetam. A CT scan of the maxillofacial region showed multiple minimally displaced acute fractures of the bilateral nasal bones with leftward deviation. A CT scan of the head showed old lacunar infarcts and asymmetric hypoattenuation in the right frontal lobe. A CT scan of the cervical spine was normal. Her lacerations were sutured, and ENT advised conservative management of her nasal fractures. She was admitted to the intensive care unit for diabetic ketoacidosis (DKA).

**Figure 1:**
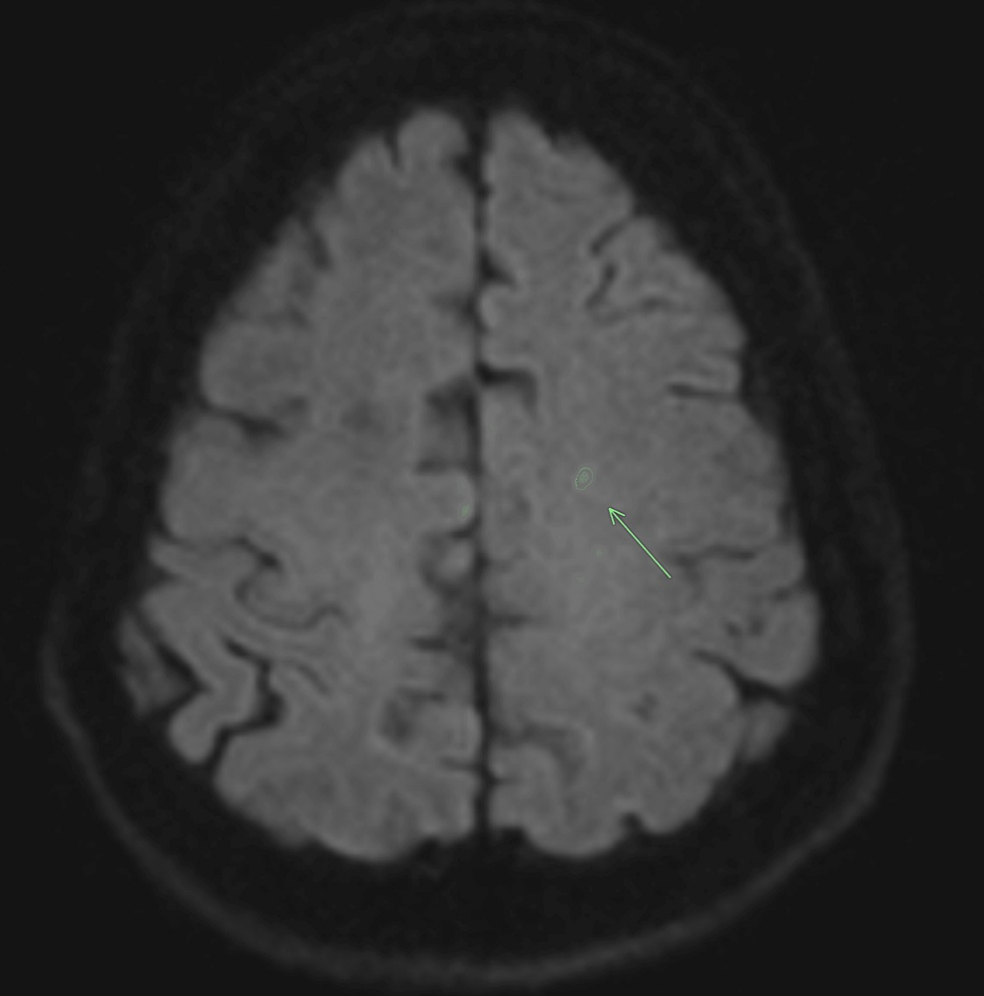
MRI brain without contrast showing a new lacunar infarct in the left frontal lobe.

We attributed her seizures to metabolic abnormalities from DKA. The EEG showed left-frontal cerebral dysfunction with epileptic potential. The MRI brain (T2 weighted) without contrast showed a new lacunar infarct in the left frontal lobe (Figure 1).

**Figure 2:**
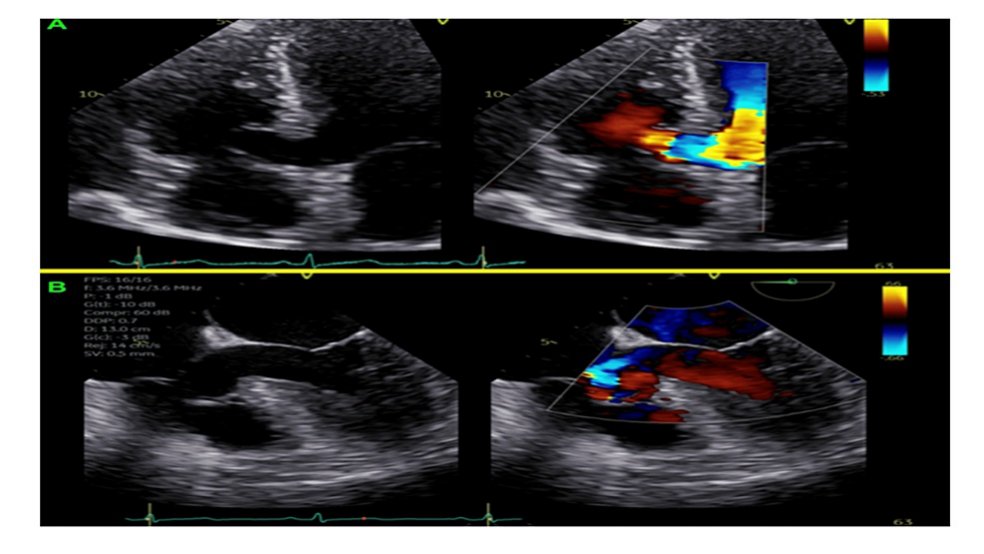
(A)Transthoracic echocardiogram revealing a turbulent flow within basal interventricular septal aneurysm without intracardiac shunting. (B) Trans-esophageal echocardiogram demonstrating a basal IVS aneurysm measuring 12.2 mm × 16 mm without intracardiac shunting or thrombi.

A transthoracic echocardiogram revealed a basal IVS aneurysm without shunting (Figure 2A). A trans-esophageal echocardiogram confirmed a basal IVS aneurysm measuring 12.2 mm × 16 mm without intracardiac shunting or thrombi (Figure 2B).

The subsequent review of prior echocardiograms showed a subtle abnormality that seemed to have enlarged over time. Medical management was recommended by cardiac surgery and cardiology at that time. She was initiated on anticoagulation and discharged on Rivaroxaban and aspirin for her strokes. Her diabetes and seizures were also managed appropriately. Apart from diabetes mellitus and congenital heart anomalies, she did not have any other risk factors for stroke. The extended hereditary thrombophilia screening work-up, including protein S (PS), protein C (PC), anti-thrombin (AT) III, Laiden factor-V mutation, factor-II levels, and the anti-phospholipid antibody panel (APLA), was unremarkable. The CTA neck did not show any evidence of significant vascular abnormalities. We implanted a loop recorder to detect arrhythmia as a possible source of embolism in her strokes. She is scheduled for an outpatient cardiology follow-up. 

## Discussion

The bicuspid aortic valve is an uncommon congenital cardiac anomaly that affects 1.3% of the general population worldwide and severe outcomes include regurgitation, ascending aneurysms, and worsening aortic stenosis [5]. The risk of cardioembolic stroke in BAV with normal valve function is very low, but severe valvular dysfunction due to severe aortic stenosis can cause embolism and its complications, but the exact mechanism is unknown. Our patient had severe aortic stenosis from BAV, increasing her risk of cardioembolic stroke from BAV [5].

IVS aneurysms are very rare, commonly benign, and clinically insignificant. IVS aneurysms can be classified as true, false, or pseudo-aneurysms. True aneurysms have a regular shape and broad base; false ones have an irregular shape and narrow base; and pseudo-aneurysms are usually secondary to a previous ischemic event. Traditionally, IVS aneurysms have been diagnosed incidentally in the setting of intra-cardiac shunts (left-to-right), embolic phenomena, endocarditis, aneurysm rupture, or RVOT obstruction. With the advent of noninvasive and highly sensitive diagnostic imaging such as echocardiography, MRI, and CT angiography, the detection rate of IVS aneurysms has increased dramatically [6].

Most patients with BAV and basal IVS aneurysms have a silent clinical course and do not need intervention. They should be monitored periodically with noninvasive techniques such as echocardiography for disease progression and the risk of complications, including cerebrovascular events. When there are associated hemodynamic changes, severe stenosis, or IVS-related complications, surgical intervention is warranted [7]. They recommended starting anticoagulation if there is a concern for thromboembolism within the ventricular septal aneurysm [8,9]. Occasionally, cerebral embolic phenomena in the setting of failed anticoagulation also warrant surgical intervention. Our patient received anticoagulation and did not qualify for surgery.

This case illustrates several clinical issues, including the need for a multidisciplinary approach to surveillance and management in managing repaired CHD like BAV and IVS aneurysms and the potential complications of these rare conditions. More than 1 million adults in the United States have CHD [8,9]. Although adults with congenital heart defects (CHD) are a more vulnerable population, they may remain undiagnosed or lose follow-up care during the transition from adolescence to adulthood. Awareness of the association between BAV and IVS aneurysms can facilitate the identification of the same. Our patient did not have regular follow-up, which probably delayed her diagnosis and led to thromboembolism.

## Conclusions

This report emphasizes that IVS aneurysms associated with BAV, although often asymptomatic, may cause adverse outcomes such as cardioembolic stroke. Therefore, timely detection by non-invasive imaging, including echocardiography, CT scans, and MRI, and appropriate intervention are essential to improving health outcomes and survival.
